# Hyperacute Serum and Knee Osteoarthritis

**DOI:** 10.7759/cureus.53118

**Published:** 2024-01-28

**Authors:** Ashim Gupta, Adarsh Aratikatla

**Affiliations:** 1 Orthopaedics and Regenerative Medicine, Regenerative Orthopaedics, Noida, IND; 2 Regenerative Medicine, Future Biologics, Lawrenceville, USA; 3 Regenerative Medicine, BioIntegrate, Lawrenceville, USA; 4 Orthopaedics, South Texas Orthopaedic Research Institute, Laredo, USA; 5 Medicine, The Royal College of Surgeons in Ireland, Dublin, IRL

**Keywords:** serum from platelet-rich fibrin, platelet-rich fibrin, platelet-rich plasma, hyperacute serum, blood-derived biologics, autologous blood, orthobiologics, regenerative medicine, osteoarthritis, knee osteoarthritis

## Abstract

The knees are the most frequently affected weight-bearing joints in osteoarthritis (OA), impacting millions of people globally. With increasing life spans and obesity rates, the prevalence of knee OA will further mount, leading to a significant increase in the economic burden. The usual treatment modalities utilized to manage knee OA have shortcomings. Over the last decade, the field of regenerative medicine involving the use of biologics, such as autologous peripheral blood-derived orthobiologics, including hyperacute serum (HS), has evolved and shown potential for managing knee OA. In this manuscript, we qualitatively present the in vitro, pre-clinical, clinical, and ongoing studies investigating the applications of HS in the context of knee OA. Seven in vitro studies and one clinical study fit the scope of our manuscript. The results demonstrated that the administration of HS is potentially safe and efficacious in terms of increasing the viability of osteoarthritic chondrocytes, reducing pain and inflammation, and improving function in patients with knee OA. However, due to insufficient literature, pre-clinical studies to better understand the mechanism of action are required. In addition, adequately powered, multi-center, non-randomized, and randomized controlled trials with longer follow-up are warranted to establish the safety and efficacy of HS for the management of knee OA and to justify its clinical use.

## Introduction and background

Osteoarthritis (OA) of the knee is the predominant joint disease, affecting millions of people worldwide [1]. Its etiology encompasses synovial tissue inflammation and worsening of the articular cartilage, leading to pain, reduced function, and impacted overall quality of life [2]. Traditionally, knee OA is managed via non-pharmacological modalities such as activity modification, weight reduction, and physiotherapy; nutraceuticals such as symptomatic slow-action drugs for OA, for example, glucosamine, chondroitin, and undenatured type II collagen; pharmacological agents such as non-steroidal anti-inflammatory drugs and opioids, and intraarticular administration of corticosteroids and viscosupplementation; minimally invasive procedures such as genicular nerve radiofrequency ablation; and surgical interferences, in later stages or after conservative approaches have been ineffective [1-4]. These aforesaid therapies have limitations and side effects, continually aiming to decrease pain rather than targeting the causal pathophysiology [1-4].

Over the last decade, there has been a prominent rise in the use of orthobiologics, including autologous peripheral blood-derived orthobiologics (APBO) for musculoskeletal regenerative medicine [4-6]. Platelet-rich plasma (PRP) is the most extensively used APBO, yet its effectiveness remains controversial, ascribed to a lack of standardized preparation protocol, inter- and intra-patient variables, etc. [4-6]. Clinicians and scientists have investigated the utilization of platelet-rich fibrin (PRF) to circumvent the limitations posed by PRP, attributed to its ability to secrete growth factors and cytokines for a longer duration compared to PRP; however, its three-dimensional (3D) structure deters its administration as an injectable [7-9]. To overcome this, hyperacute serum (HS), also known as serum from PRF, was developed [10]. HS is a cell-, platelet-, and fibrin matrix-free formulation prepared by squeezing the serum from the formed PRF clot [10]. Up until now, only a limited number of studies have investigated the safety and efficacy of HS for the treatment of knee OA. The primary objective of this study is to review the in vitro, pre-clinical, and clinical outcomes of HS for the treatment of knee OA. The secondary objective is to document the ongoing clinical trials registered on different trial protocol repositories related to HS for the management of knee OA.

## Review

Search criteria

**Figure 1:**
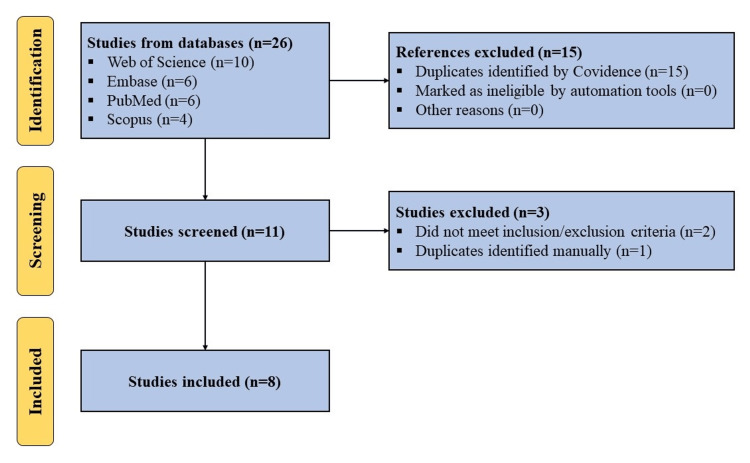
PRISMA flow diagram outlining the record identification and selection process PRISMA: Preferred Reporting Items for Systematic Reviews and Meta-analyses

A search was conducted using Web of Science, Embase, PubMed, and Scopus for articles published in English before January 2024 regarding the use of HS for the treatment of knee OA. We adhered to the Preferred Reporting Items for Systematic Reviews and Meta-analyses (PRISMA) statement and guidelines. The following search terms: ("knee") AND ("osteoarthritis" OR "OA") AND ("hyperacute serum") were utilized. All in vitro, pre-clinical, and clinical studies were included. Studies not utilizing HS as an intervention or not focusing on the management of knee OA were excluded (Figure 1).

We also searched the Chinese Clinical Trial Register (ChiCTR), Clinical Trials Registry - India (CTRI), and ClinicalTrials.gov using the abovementioned search terms to find ongoing trials registered on these repositories related to the use of HS for the treatment of knee OA.

Results

In Vitro Studies

Simon et al. investigated the effect of HS on monolayer mesenchymal stem cell (MSC) cultures and in 3D subchondral bone and marrow explants. The viability measurements, real-time quantitative polymerase chain reaction (RT-qPCR) for expression of genes, and flow cytometry (FACS) for cell surface marker testing were conducted to determine the effect of supplementing culture media with HS compared to the PRP and fetal calf serum (FCS) [11]. The supplementation with HS led to significantly higher cell proliferation at Day 5 compared to the baseline and other groups. FACS showed that MSC markers were retained post-incubation with HS, similar to the FCS group, but variations were noted in the PRP group. RT-qPCR confirmed no change in MSC markers post-incubation with HS. Supplementation with HS also led to significantly higher expression levels of osteogenic gene markers. A similar effect was observed in bone and marrow explants. In addition, histology analysis showed higher preservation of bone marrow integrity with HS supplementation compared to the FCS. Additionally, Simon et al. also investigated the effect of HS on osteoarthritic chondrocytes [11]. The results showed significant improvement in viability in the HS-incubated group compared to the FCS and PRP groups on Days 4 and 8. In summary, the findings from this study demonstrated the potential of HS for musculoskeletal regenerative medicine applications, including knee OA.

Kardos et al. investigated the effect of HS supplementation on the cytokine profile of a complex OA joint in an in vitro model [12]. Hyaline cartilage, subchondral bone, and synovial membrane were harvested from osteoarthritic knees during regular knee replacement surgery, cultured, and stimulated with interleukin-1β (IL-1β). This is then treated with either 10% HS or human serum albumin for five days. Tissues cultured without stimulation with IL-1β were considered a negative control. Synovial fluid was also aspirated from individuals who underwent intra-articular knee injection therapies. The concentration of 39 cytokines, known to play a role in OA, was measured in both the culture supernatant after IL-1β treatment and in the synovial fluid. The Pearson correlation test confirmed a strong correlation between the in vitro and in vivo samples, signifying that the in vitro model reasonably mimics the in vivo knee OA model in terms of cytokine profile. HS led to significantly higher cell viability in the cartilage, bone, and synovial membrane. The treatment with HS led to a significant decrease in the expression of inflammatory cytokines including IL-1β, interleukin-6 receptor-α (IL-6Rα), and tumor necrosis factor-α (TNF-α), and a significant increase in the levels of anti-inflammatory interleukin-1 receptor antagonist (IL-1RA). In addition, HS treatment resulted in higher expression levels of osteonectin, released by regenerating osteoblasts, chondrocytes, and bone marrow progenitor cells, and collagen type I alpha I (Col1A1), secreted by osteoblasts and responsible for forming 90% of the bone extracellular matrix. Additionally, the levels of osteoclastogenesis and bone resorption inducing RANKL were significantly reduced post-treatment with HS. The results from this study demonstrated the potential of HS to induce cell proliferation and change the microenvironment toward a less inflamed state in the osteoarthritic knee joint.

Neubauer et al. investigated the effect of HS compared to EPRP (PRP prepared in the presence of EDTA) and CPRP (PRP prepared in the presence of citrate) on the differentiation potential of adipose-derived MSCs (AD-MSCs) from three fat locations, namely, Hoffa’s/infrapatellar fat pad (IFP), distal femur/pouch (prefemoral/supratrochlear) fat, and subcutaneous fat, isolated during knee replacement procedure [13]. Isolated AD-MSCs were analyzed via FACS. Cells were supplemented with HS, EPRP, CPRP, and FCS (positive control), and cell viability, expression of genes, and differentiation were determined. EPRP and CPRP formulations prepared were leukocyte-poor PRP. FACS showed the presence of MSC markers and the absence of hematopoietic stem cell markers on cells isolated from all three fat locations. The HS and CPRP groups showed significantly higher metabolic activity compared to the EPRP and FCS groups on Day 6, while no differences were observed between the HS and CPRP groups. EPRP and CPRP led to enhanced chondrogenesis and osteogenesis, respectively, while HS led to enhanced differentiation towards both lineages. Interestingly, both PRP types used showed varying biological effects in terms of chondrogenic gene expression depending on the concentration used, though no such variation was observed with HS supplementation. The results from this study demonstrated that the type of anti-coagulant and concentration of PRP significantly affect the differentiation potential of AD-MSCs, whereas cell-free, anti-coagulant-free HS led to no such variability and, thus, has the potential to be used for regenerative medicine applications, including for OA knee.

Otahal et al. investigated the role of extracellular vesicles (EVs) isolated from HS and CPRP in an inflammation model where patient-derived primary OA chondrocytes were co-cultured with activated M1 macrophages [14]. EVs were isolated from HS and CPRP via ultracentrifugation and characterized via western blotting and nanoparticle tracking analysis. OA chondrocytes were isolated from cartilage harvested from individuals undergoing knee replacement surgery. Primary monocytes were acquired from healthy donors and were differentiated and activated into pro-inflammatory M1 macrophages. RT-qPCR results showed significantly increased expression of collagen type II and aggrecan post-treatment with EVs isolated from HS and HS, respectively. In addition, ELISA showed lower expression levels of pro-inflammatory TNF-α and IL-1β post-treatment with EVs isolated from HS. The results from this study indicated the potential of EVs from HS to be used as therapeutic agents for the management of knee OA.

Kuten-Pella et al. assessed and compared the regenerative potential of HS, EPRP, and CPRP using an osteoarthritic chondrocyte 3D pellet model by evaluating the metabolic activity of cells and the expression of genes within the pellet [15]. No significant differences in metabolic activity were observed between the three groups. HS treatment led to significant improvements in the expression of chondrogenic-related genes, SOX-9 and COL2AB, at Day 21 compared to Day 0. Both PRP types had different biological effects on gene expression depending on the concentration used, though no such variation was observed with the HS supplementation. In summary, the results from this study demonstrated the ability of HS to enhance cell proliferation and expression of chondrogenic-related genes, indicating the potential of HS to be used for the management of knee OA.

Calvo et al. investigated the regenerative potential of lyophilized HS and HS in combination with hyaluronic acid (HA) [16]. OA chondrocytes were isolated from cartilage obtained from patients undergoing knee replacement surgery and tested for viability of cells and expression of genes related to OA. An explant co-culture comprising cartilage, bone, and synovial membrane subjected to inflammatory conditions was utilized to determine the anti-inflammatory ability of lyophilized HS and HS+HA. The lyophilized HS led to a significantly higher rate of cell proliferation compared to the baseline, and the effect was similar to freshly prepared HS or PRP formulations. No significant differences were observed between lyophilized and freshly prepared HS formulations. RT-qPCR showed significantly higher expression of Col1A1 post-treatment with both fresh and lyophilized HS compared to the PRP. In addition, HS supplementation resulted in significantly decreased expression of pro-inflammatory cytokines, including IL-2, IL-5, IL-7, IL-12, IL-15, and TNF-α. These results demonstrated the ability of HS to enhance cell proliferation and reduce inflammation, thereby indicating its potential to be used as a therapeutic agent for the management of knee OA.

Neubauer et al. investigated the effect of HS compared to PRP on IFP-MSC viability and chondrogenic differentiation [17]. FACS was used to determine cell viability and MSC markers. Metabolic activity via the XTT assay and chondrogenic differentiation via Alcian blue staining were determined. RT-qPCR was used to determine the expression of cartilage-specific gene markers. FACS showed the presence of over 80% viable cells, and these cells expressed markers for MSCs. The metabolic activity was significantly higher in the PRP and HS-treated cells compared to the baseline and control FCS, while no significant differences were reported between the HS and PRP groups. All groups showed an increase in the expression of cartilage-specific genes, but the increase in the expression of collagen type II and aggrecan was significantly higher for the HS group. Histological analysis via Alcian Blue stain confirmed the differentiation of isolated MSCs toward the chondrogenic lineage in all groups. The results from this study showed the potential of these isolated MSCs for regenerative medicine applications, and augmentation with HS can lead to their enhanced proliferation and differentiation towards chondrogenic differentiation, essential for cartilage regeneration in the OA knee. The results from in vitro studies are summarized in Table 1.

**Table 1:** Summary of results of included in vitro and clinical studies HS: hyperacute serum, MSCs: mesenchymal stem cells, PRP: platelet-rich plasma, FCS: fetal calf serum, EPRP: PRP prepared in the presence of EDTA, CPRP: PRP prepared in the presence of citrate, AD-MSCs: adipose-derived MSCs, 3D: three dimensional, IFP: infrapatellar fat pad, EVs: extracellular vesicles, VAS: visual analogue scale, KOOS: knee injury and osteoarthritis outcome score

Author	Reference	Study type	Main findings
Simon et al.	[11]	In vitro	Supplementation with HS led to significantly higher viability of MSCs, and bone and marrow explants along with higher integrity of bone marrow. In addition, it also led to significantly higher viability of osteoarthritic chondrocytes compared to PRP and FCS.
Kardos et al.	[12]	In vitro	Supplementation with HS led to significantly higher cell viability of cartilage, bone, and synovial membrane. It also led to a significant decrease in the expression of pro-inflammatory cytokines and an increase in the expression of anti-inflammatory cytokines.
Neubauer et al.	[13]	In vitro	Supplementation with HS led to significantly higher viability of AD-MSCs isolated from 3 different tissue types compared to EPRP and FCS. It also led to enhanced differentiation towards both osteogenic and chondrogenic lineages, unlike EPRP and CPRP which showed varied outcomes in terms of concentration used and differentiation towards specific lineage.
Otahal et al.	[14]	In vitro	EVs isolated from HS led to significantly higher expression of chondrogenic-lineage specific collagen type II and reduced expression of pro-inflammatory cytokines.
Kuten-Pella et al.	[15]	In vitro	Supplementation with HS led to significantly higher expression of chondrogenic-related genes in a 3D osteoarthritic chondrocyte pellet model.
Calvo et al.	[16]	In vitro	Supplementation with lyophilized HS led to a significantly higher rate of cell proliferation, similar to that of freshly prepared HS. In addition, it led to a significant reduction in the expression levels of pro-inflammatory cytokines.
Neubauer et al.	[17]	In vitro	Supplementation with HS led to the significantly higher metabolic activity of AD-MSCs isolated from the IFP. It also led to a significant increase in the expression of chondrogenic-lineage-specific genes.
Calvo et al.	[18]	Clinical	Intra-articular administration of 3 mL autologous HS (three times on a weekly basis) led to significant improvements in pain measured via VAS, and mobility/function measured via KOOS and Lysholm-Tegner score at six months follow-up compared to the baseline.

Pre-clinical Studies

Thus far, there are no published pre-clinical studies concerning the use of HS for the management of knee OA.

Clinical Studies

Calvo et al. evaluated the safety, feasibility, and effectiveness of HS in knee OA patients [18]. The inclusion criteria included ≥18 and ≤70 years old males or females, the presence of Grade II or III OA (on the Kellgren-Lawrence scale) in the index knee, the pain of at least 4 (on a 10-point scale) on the visual analogue scale (VAS), etc. The exclusion criteria included patients with BMI >40, intra-articular therapy in the index knee in the last six months, systemic use of steroids in the last three months, other non-OA joint disease, etc. 24 patients met the inclusion/exclusion criteria and were intra-articularly injected three times on a weekly basis with 3 mL of autologous HS. The patient-reported outcome measures (PROMs) included VAS for pain, Lysholm-Tegner for a knee injury, and the osteoarthritis outcome score (KOOS) for mobility. The PROMs were recorded at baseline and at one week, two weeks, three months, and six months of follow-up. The included patients were also divided into two subgroups: patients with effusion and patients without effusion. No adverse events were reported throughout the duration of the study. HS demonstrated a steady improvement in terms of knee instability, pain, swelling, stair climbing and squatting, and mechanical locking, as assessed by the Lysholm-Tegner score over time. This improvement was statistically significant at six months compared to the baseline for both groups, though no differences between the two groups were observed. A similar positive outcome was reported for both VAS and KOOS scores. The main shortcomings of this study are the short follow-up, small sample size, and absence of a control group(s). Despite these, the results of this study showed the potential of HS in reducing pain and improving function in patients suffering from knee OA. The results from this study are summarized in Table 1.

Ongoing Clinical Studies

As of January 14, 2024, there are no ongoing clinical studies registered on ChiCTR, CTRI, or ClinicalTrials.gov to study the safety and efficacy of HS for the management of knee OA.

Discussion

The present study evaluated the therapeutic potential of HS for the management of knee OA. In vitro, pre-clinical, and clinical studies focusing on the effect of HS on knee OA were included. Based on our search criteria and inclusion/exclusion criteria, seven in vitro and one clinical studies fit the scope of our manuscript. No pre-clinical studies were found. This could be attributed to the relatively new discovery of HS compared to other APBOs. In addition, no ongoing clinical trials were registered on different clinical trial protocol registries.

The in vitro studies demonstrated that treatment with HS resulted in increased viability of osteoarthritic chondrocytes and MSCs, reduced expression of pro-inflammatory cytokines, increased expression of anti-inflammatory cytokines, increased differentiation of MSCs towards the chondrogenic lineage, and increased expression of chondrogenesis-related genes [11-17]. In addition, the cell-free and anti-coagulant-free nature of HS led to no variability in the differentiation potential or gene expression of MSCs, unlike PRP. Additionally, the identified clinical study, one of the first, demonstrated that the administration of HS is potentially safe and efficacious in terms of reducing pain and improving function in patients suffering from knee OA [18].

## Conclusions

Notwithstanding methodological limitations and the dearth of relevant literature, these studies laid the groundwork for future pre-clinical studies to better understand the mechanism of action and for adequately powered, multi-center, non-randomized, and randomized controlled trials with longer follow-up along with post-market real-world studies in clinical practice to establish the safety and efficacy of HS in patients with knee OA. In the future, more clinical studies assessing the effectiveness of HS compared to other APBOs are also warranted to aid clinicians in determining the most optimal APBO for the management of knee OA.
